# Insolation and Disease Severity in Paediatric Inflammatory Bowel Disease—A Multi-Centre Cross-Sectional Study

**DOI:** 10.3390/jcm9123957

**Published:** 2020-12-07

**Authors:** Aleksandra Glapa-Nowak, Mariusz Szczepanik, Jarosław Kwiecień, Anna Szaflarska-Popławska, Anna Flak-Wancerz, Barbara Iwańczak, Marcin Osiecki, Jarosław Kierkuś, Tomasz Pytrus, Dariusz Lebensztejn, Tomasz Banasiewicz, Aleksandra Banaszkiewicz, Jarosław Walkowiak

**Affiliations:** 1 Department of Pediatric Gastroenterology and Metabolic Diseases, Poznań University of Medical Sciences, 60-572 Poznan, Poland; glapa@ump.edu.pl (A.G.-N.); mszczepanik@ump.edu.pl (M.S.); 2 Department of Pediatrics, Faculty of Medical Sciences in Zabrze, Medical University of Silesia, 41-800 Katowice, Poland; jkwiecien@sum.edu.pl; 3 Department of Pediatric Endoscopy and Gastrointestinal Function Testing, Collegium Medicum in Bydgoszcz, Nicolaus Copernicus University in Toruń, 85-067 Bydgoszcz, Poland; aszaflarska@wp.pl; 4 Department of Pediatrics, Faculty of Medical Sciences, Medical University of Silesia in Katowice, 40-752 Katowice, Poland; fitmed@wp.pl; 5 Department and Clinic of Pediatrics, Gastroenterology and Nutrition, Wroclaw Medical University, 50-369 Wroclaw, Poland; Barbara.iwanczak@umed.wroc.pl (B.I.); tomasz.pytrus@umed.wroc.pl (T.P.); 6 The Department of Gastroenterology, Hepatology, Feeding Disorders and Pediatrics, The Children’s Memorial Health Institute, 04-730 Warsaw, Poland; m.osiecki@ipczd.pl (M.O.); j.kierkus@med-net.pl (J.K.); 7 Department of Pediatrics, Gastroenterology, Hepatology, Nutrition and Allergology, Medical University of Bialystok, 15-274 Bialystok, Poland; lebensztejn@hoga.pl; 8 Department of General and Endocrine Surgery and Gastroenterological Oncology, Poznań University of Medical Sciences, 60-355 Poznan, Poland; tbanasie@ump.edu.pl; 9 Department of Pediatric Gastroenterology and Nutrition, Medical University of Warsaw, 02-097 Warsaw, Poland; aleksandra.banaszkiewicz@wum.edu.pl

**Keywords:** seasonality, seasonal variation, Crohn’s disease, ulcerative colitis, treatment, surgery

## Abstract

This study was to investigate whether the clinical course of inflammatory bowel disease (IBD) in a Polish paediatric cohort fits a seasonal pattern and depends on insolation. Two hundred and fourteen patients diagnosed with Crohn’s disease (CD) and 192 with ulcerative colitis (UC) aged from 3 to 18 years, were recruited in seven centres of similar latitude. The seasons were defined as winter (December–February), spring (March–May), summer (June–August), autumn (September–November). The year was also divided depending on insolation threshold (3.0 kWh/m^2^/day). Patients diagnosed with IBD when the isolation was >3 kWh/m^2^/day had poorer nutritional status than those diagnosed while insolation was below threshold (lower standardised BMI at diagnosis (−0.81 ([−1.34]–[−0.03]) vs. −0.52 ([−1.15]–0.15); *p* = 0.0320) and worst flare (−0.93 ([−1.37]–[−0.05]) vs. −0.66 ([−1.23]–0.17); *p* = 0.0344), with the need for more frequent biological treatment (45.5% vs. 32.7%, *p* = 0.0100). Patients diagnosed in winter were significantly younger at diagnosis (11.4 vs. 13.0; *p*_adj_ = 0.0180) and first immunosuppressive treatment (11.3 vs. 13.3; *p*_adj_ = 0.0109) than those diagnosed in other seasons. CD patients diagnosed in months with higher insolation spent more days in hospital than those diagnosed in months with lower insolation [4.6 (1.8–11.8) vs. 2.9 (1.3–6.2); *p* = 0.0482]. CD patients diagnosed in summer had significantly more concomitant diseases. In patients with CD, the occurrence of the worst flare was more frequent in autumn. Furthermore, the season of birth was associated with Pediatric Crohn’s Disease Activity Index at worst flare and earlier surgery. In conclusion, several clinical parameters are associated with insolation, the season of diagnosis and season of birth in the clinical course of Crohn’s disease.

## 1. Introduction

Inflammatory bowel disease (IBD) is a chronic gastroenterological disorder, with ulcerative colitis (UC) and Crohn’s disease (CD) being the most prevalent diseases [1]. However, their aetiology is unclear, with unpredictable patterns of relapses and remissions [1]. Predicting benign or unfavourable clinical course is crucial to avoid overtreatment and facilitate clinical decisions. Recently, reduced vitamin D levels in IBD were associated with clinical relapse, increased disease activity and inflammation [2,3]. This deficiency is thought to be related to malnutrition, inadequate dietary intake, lower bioavailability as well as reduced sunlight exposure. Higher sun exposure in the previous summer or winter was associated with a reduced risk of developing IBD [4].

The seasonal variations in IBD have been of interest to researchers, however, the evidence regarding the concept of seasonal patterns is contradictory. The variation in results may be caused by a different definition of flares, geographical and genetic background or environmental factors such as infections, climate, and diet [5]. UC has been reported to run a clinical course with seasonal exacerbations in retrospective studies [6]. Seasonal change has also been observed later in CD [7], showing the highest relapse rate in the autumn and winter, with the lowest in summer. However, contradictory studies have reported increased IBD flares in spring [8,9]. Low sun exposure was associated with longer hospitalisations and bowel surgeries [10]. 

The present study investigated the associations of the season of birth, season and the level of insolation at diagnosis with disease characteristics in a paediatric IBD cohort, using nutritional status, C-reactive protein levels (CRP), disease activity scales, number of systemic steroid courses, biologics, immunosuppressants and surgeries with age and time to aforementioned treatments. We also compared numbers of relapses, hospitalisations and days spent in hospital, crucial from the patient’s and health care perspective. 

## 2. Experimental Section

Patients were recruited in seven Polish centres (POCOCO cohort—Polish Paediatric Crohn’s and Colitis cohort) (Table 1). The recruitment of patients took place in the Department of Paediatric Gastroenterology and Metabolic Diseases Poznań University of Medical Sciences, The Department of Gastroenterology, Hepatology, Feeding Disorders and Paediatrics, The Children’s Memorial Health Institute, Warsaw; Department of Paediatric Gastroenterology and Nutrition, Medical University of Warsaw; Department and Clinic of Paediatrics, Gastroenterology and Nutrition, Wroclaw Medical University; Department of Paediatrics, Faculty of Medical Sciences in Zabrze, Medical University of Silesia, Katowice; Department of Paediatrics, Faculty of Medical Sciences, the Medical University of Silesia in Katowice and Department of Paediatric Endoscopy and Gastrointestinal Function Testing, Collegium Medicum in Bydgoszcz, Nicolaus Copernicus University in Toruń, Bydgoszcz. The diagnosis was established between 2003–2019 based on clinical, histological, endoscopic, and/or radiological criteria. All centers were operating according similar, accepted guidelines. Disease localisation and behaviour were determined according to the Paris Classification (for CD: L1: Ileal; L2: Colonic; L3: Ileocolonic; L4a: upper disease proximal to ligament of Treitz; L4b: upper disease distal to ligament of Treitz; B1: nonstricturing; B2: structuring; B3: penetrating; B2B3: penetrating and structuring; P: perianal disease modifier. For UC: E1: Proctitis; E2: Left-sided colitis; E3: Extensive colitis; E4: Pancolitis; S0: never severe; S1: ever severe). Disease activity was defined with Pediatric Crohn’s Disease Activity Index (PCDAI) and Paediatric Ulcerative Colitis Activity Index (PUCAI). Clinical relapse was defined as an increase in symptom intensity and inflammatory markers that lead to the intensification in disease management or hospitalization. The criteria to initiate the biological treatment were as per drug programs’ regulations defined by Polish Ministry of Health, uniform for all centers. Steroids were used according to contemporary European guidelines. 

The date of diagnosis was established according to the date or month of onset of symptoms when the diagnosis of IBD could be defined. Exclusion criteria were the lack of data on the timing of diagnosis and life-threatening, severe general condition (in severe pain or distress, awaiting urgent surgery or immediately afterwards, with severe anemia). The inclusion criteria were a diagnosis of CD or UC and aged 3–18. The date of exacerbation was obtained by reviewing records from outpatient visits, hospitalisations, emergency department visits and telephone encounters. The month of birth recorded in the hospital database was used to determine the season of birth. The seasons were defined as winter (December–February), spring (March–May), summer (June–August), autumn (September–November). The insolation was defined as the average amount of the total solar radiation incident on a horizontal earth surface for a given month, averaged for that month over the 22-year period (July 1983–June 2005). Each monthly averaged value is evaluated as the numerical average of 3-hourly values for the given month based on source: NASA Langley Research Center Atmospheric Science Data Center. The monthly insolation in central Poland e.g., January: 0.87; February: 1.54; March: 2.55; April: 3.72; May: 4.97; June: 4.77; July: 4.83; August: 4.39; September: 2.80; October: 1.62; November: 0.92; December: 0.67 [kWh/m^2^/day]. The insolation threshold was set at 3.0 kWh/m^2^/day according to www.gaisma.com (months >3.0 kWh/m^2^/day: April–August). Based on data from the study on Polish children [11] we measured clinical parameters in CD during seasons with typically the highest (July–October) and lowest (January–April) vitamin D levels in Poland and during seasons with the highest (May–August) and lowest (November–February) insolation.

The normality of data distribution was tested with Shapiro-Wilk test. Values were presented in medians with interquartile ranges (IQR). The comparisons between two groups were performed with Mann-Whitney U test. The comparisons between seasons were performed with Kruskal Wallis test. Post hoc comparisons were performed with Dunn’s test with Holm and Bonferroni adjustment. 

Bioethical Committee approval for the study was obtained from Poznań University of Medical Sciences (960/15 with the associated amendments). 

## 3. Results

This study involved 406 IBD patients aged 3–18 (CD *n* = 214 and UC *n* = 192, F = 173, M = 233). Basic characteristics of patients are presented in Table S1 of the Supplementary Materials. Clinical characteristics of all patients depending on insolation and season at diagnosis are presented respectively in Tables S2 and S4. Patients diagnosed with CD in April–August took more biological agents during the course of their disease (Table 1). They also had lower albumin levels at diagnosis and were hospitalised longer than patients diagnosed between September and March (Table 1). There was no difference in clinical characteristics of patients with UC depending on insolation (Table S3). Patients diagnosed with CD when insolation was >3 kWh/m^2^/day tended to have more penetrating and structuring (B2B3) disease behaviour at the worst flare (2.8% vs. 0.0%, *p* = 0.0158).

Patients diagnosed with IBD when the insolation was >3 kWh/m^2^/day (diagnosed in April–August) had lower standardised body weight at diagnosis (−0.79 ([−1.36]–[−0.12]) vs. −0.52([−1.19]–0.16); *p* = 0.0293), and worst flare (−0.83 ([−1.36]–[0.14]) vs. −0.59 ([−1.2]–0.18); *p* = 0.0344) and lower standardised BMI at diagnosis (−0.81 ([−1.34]–[−0.03]) vs. −0.52 ([−1.15]–0.15); *p* = 0.0320) and worst flare (−0.93 ([−1.37]–[−0.05]) vs. −0.66 ([−1.23]–0.17); *p* = 0.0344). Patients with CD diagnosed in April–August had lower standardised body weight at diagnosis (−0.97 ([−1.44]–[0.27]) vs. −0.66 ([−1.37]–0.13); *p* = 0.0462) than patients diagnosed with CD between September and March. No such difference was present in UC patients.

The diagnosis of UC/CD was not associated with season (*p* = 0.2305) or insolation (*p* = 0.6164). Furthermore, the occurrence of the worst flare was not associated with any specific month (*p* = 0.2167), but in patients with CD, it was more frequent in autumn (autumn 39.5% vs. spring 18.4% vs. summer 21.6% vs. winter 20.5%, *p* = 0.0405). Patients with IBD who were diagnosed in spring had significantly higher frequency of concomitant diseases than those diagnosed in summer (Table S4). In CD, there were differences depending on season in albumin levels at diagnosis and frequency of concomitant diseases and extraintestinal manifestations (Table 2). There was no difference in clinical characteristics of patients with UC depending on season of diagnosis (Table S5).

In CD, penetrating disease (B3) at diagnosis occurred more frequently in autumn (autumn 11 (10.4%), spring 3 (3.5%), summer 4 (3.2%), winter 1 (1.1%)); *p* = 0.0136; Holm-corrected post-hoc *p* value <0.05 for autumn vs. each of the other seasons. Similar pattern was observed at worst flare (*p* = 0.0426; *p*_adj_ = 0.0341). There were no associations between season of diagnosis and localisation of the disease.

The season with the least biological treatment was autumn (autumn 19.2% vs. winter 29.1%, *p* = 0.0297 and autumn 19.2% vs. summer 28.5%, *p* = 0.0394). The difference was significant for CD patients only (autumn 18.4% vs. winter 30.1%, *p* = 0.0366).

Children born in autumn presented the lowest values of PCDAI at their worst flare (autumn: 33 (24–50) vs. spring: 50 (30–55) vs. winter: 50 (37–53) vs. summer: 40 (30–52), *p* = 0.0277, post hoc comparisons: spring vs. autumn *p* = 0.0307 (Bonferroni and Holm)). Also, children born in winter had their surgeries earlier [months] in the disease course [winter: 3.0 (0.0–5.8) vs. spring: 36.8 (11.7–42.5) vs. summer 22.0 (10.1–49.1) vs. autumn 27.8 (24.1–35.2), *p* = 0.0471; post hoc comparisons: spring vs. winter *p* = 0.0393 (Holm and Bonferroni)].

We observed that in months with typically low and high vitamin D concentrations in Polish children, the number of days spent in hospital due to relapse and frequency of extraintestinal manifestations in CD show differences (Table 3).

## 4. Discussion

The clinical data regarding the association between IBD and insolation are scarce, although seasonality in IBD has been discussed several times. The present study is one of the largest to show cross-sectional associations of insolation with disease phenotype in a paediatric multi-centre cohort. We found that patients diagnosed with IBD when the insolation >3 kWh/m^2^/day (April–August) differed in nutritional status than those diagnosed between September and March, which is in line with previous data in healthy children [12]. Patients diagnosed between April and August also had more frequent biological treatment, whereas patients diagnosed in winter were significantly younger at diagnosis and first immunosuppressive treatment than those diagnosed in other seasons. CD patients diagnosed in April-August spent more days in hospital than those diagnosed between September and March, with those diagnosed in summer having significantly more concomitant diseases. In patients with CD, the occurrence of the worst flare was more frequent in autumn. Season of birth might be associated with PCDAI at worst flare and earlier surgery. 

Previous data has already shown an increase of IBD flares in the autumn/winter months compared to spring [6,7]. Riley et al. showed a seasonal pattern in UC relapse, with more relapses from August to January [6]. A few years later, similar findings were reported for CD, with the highest relapse rate in the autumn and winter [7]. In the present study, only patients with CD had their worst flares more frequently in autumn. These seasonal variations may be partially explained by sunlight exposure and vitamin D activation. In our study, we did not find any associations with the season and disease diagnosis, whereas in a Japanese study, UC onset was reported most frequently during the winter and relapses commonly occurred in October [13]. A recent study reported that winter was the most common season for disease exacerbation in both CD and UC patients [5]. However, few studies reported converse results. In a large retrospective study, Lewis et al. reported a rise in IBD flares in spring [8]. Similarly, in a Chinese study, the peak number of flares occurred during the spring and summer, especially in June [9]. The variation in results may be caused by a different definition of flares, different geographical and genetic backgrounds of patients and environmental factors such as infections, climate, and diet [5].

In the study of Peng, the frequency of onset and relapse in IBD showed seasonality only in CD, with a peak in July and August [14]. Indeed, in our study, we observed more days of hospitalisation due to relapse in the months with higher insolation (April-August). Similarly, Ahmed et al. also reported a non-significant increase in exacerbations and hospitalisation rate in July and August [15]. This seems to be in contrast with the studies showing that lower ultraviolet exposure is associated with greater rates of hospitalisation, prolonged hospitalisation and the need for surgery in IBD [10,16,17].

Taken together, the seasonal pattern alone is not a reliable marker to predict flares, even in light of promising evidence [18]. Most studies lack clinical characteristics and their association with seasons. The strength of our study is the comprehensive clinical characteristics employed to attempt to define the disease severity. Nevertheless, the study is cross-sectional, so causality cannot be determined. It would be interesting to investigate vitamin D levels to explain the associations of the season with severity. However, the seasonal changes of 25(OH)D concentrations in Polish children has been well documented earlier [11,19]. In the study of Smyczyńska el al. (*n* = 1275) the highest concentrations of 25(OH)D were observed in August (incidence of deficiency was 10.7%) and minimal in January (incidence of deficiency was 80.4%) [19]. As for seasons the highest values were in summer—July, August, September, and lowest in winter—January, February, March, with no difference between autumn and spring [19]. The level of insolation in previous months remained in close relationship with 25(OH)D levels and in Polish children a two-month shift was observed between the highest insolation and maximal 25(OH)D level [19]. This is due to long half-life of serum 25(OH)D. Indeed, the highest insolation in Poland is observed in May (4.97 kWh/m^2^/day) and the lowest in December (0.67 kWh/m^2^/day), which confirms the two-month shift. In the study of Chlebna-Sokół et al. the lowest mean concentration of 25(OH)D were observed in February and November, whereas only in August and September the values reached optimal level (>30 ng/mL) [11]. In the remaining months children (*n* = 1007) were deficient in vitamin D [11]. In our simulation analysis based on data from previous study in Polish children, months with typically high and low vitamin D status seem to be associated with number of days spent in hospital due to relapse and frequency of extraintestinal manifestations. These results indicate that the associations of vitamin D and clinical parameters do not go hand in hand with associations observed depending on insolation levels. Looking forward, there is still much to be learned about the aetiology of IBD and how specific environmental exposure impacts the development of disease as well as the potential for relapse.

## 5. Conclusions

There were differences in the clinical expression of IBD depending on insolation and season at diagnosis in the paediatric cohort. Stronger insolation (>3 kWh/m^2^/day) was associated with poor nutritional status and a higher frequency of biological treatment. Children diagnosed in winter were younger at first immunosuppressive treatment, with CD patients diagnosed between April and August spending more days in hospital than those diagnosed in other months. Furthermore, CD patients diagnosed in summer had significantly more concomitant diseases. The worst flare of CD most frequently occurred in autumn and patients diagnosed with CD in autumn had more extraintestinal manifestations. Furthermore, the season of birth might be associated with PCDAI at worst flare and earlier surgery.

## Figures and Tables

**Table 1:** Clinical characteristics of patients with Crohn’s disease depending on insolation.

Variables Median (IQR) or n (%)	Insolation		*p* Value
	>3 kWh/m^2^/day (April–August)	<3 kWh/m^2^/day (September–March)	
**Age**			
At diagnosis	12.8 (9.1–14.5)	12.2 (10.1–14.2)	0.9478
At worst flare	13.8 (12.2–16.1)	13.5 (11.2–15.6)	0.2393
**Selected biochemical parameters**			
CRP at diagnosis [mg/L]	15.1 (2.3–34.0)	10.4 (2.0–21.0)	0.1203
CRP at worst flare [mg/L]	14.0 (3.2–37.5)	13.5 (2.2–31.2)	0.5894
Albumin level at diagnosis [g/dL]	**3.8 (3.4–4.1)**	**4.0 (3.8–4.3)**	**0.0032**
Albumin level at worst flare [g/dL]	3.9 (3.4–4.1)	3.9 (3.6–4.3)	0.3400
**Disease activity scales**			
PCDAI at diagnosis	35 (25–48)	30 (20–48)	0.1031
PCDAI at worst flare	44 (30–54)	39 (25–53)	0.2604
**Treatment**			
Number of patients on systemic steroids *	58 (59.8)	57 (48.7)	0.1299
Number of courses of steroid treatment	1 (1–2)	1 (0–2)	0.1794
Number of patients receiving immunosuppressive treatment **	77 (79.4)	91 (77.8)	0.8676
Number of immunosuppressants	1 (1–1)	1 (1–1)	0.5960
Time to first dose of immunosuppressive treatment [months]	2.2 (0.0–11.8)	1.1 (0.0–6.2)	0.8222
Age at first intake of immunosuppressive treatment [years]	12.9 (10.4–14.6)	12.8 (10.4–14.9)	0.8485
Number of patients receiving biological therapy ***	56 (43.6)	51 (57.7)	0.0543
Number of biological agents	**1 (0–1)**	**0 (0–1)**	**0.0064**
Time to first dose of biological treatment [months]	11.5 (5.9–22.7)	19.5 (6.3–32.1)	0.1218
Age at first biological treatment	13.6 (12.0–15.2)	13.8 (10.9–15.4)	0.8768
Number of patients who had operative treatment ****	14 (14.4)	15 (12.8)	0.8415
Age at first surgery [years]	14.0 (13.2–14.9)	14.6 (12.5–16.2)	0.8094
Time to first surgery [months]	23.1 (0.0–35.0)	17.5 (5.0–49.7)	0.6238
**Hospitalisations (if duration ≥1 years)**			
Hospitalisations for relapse (per 1 year)	0.5 (0.2–0.9)	0.4 (0.2–0.8)	0.3511
Days of hospitalisation for relapse (per 1 year)	**4.6 (1.8–11.8)**	**2.9 (1.3–6.2)**	**0.0482**
Relapses from diagnosis (per 1 year)	0.5 (0.2–1.1)	0.4 (0.2–0.8)	0.2647
Severe relapses from diagnosis (per 1 year)	0.2 (0.0–0.5)	0.2 (0.0–0.4)	0.7085
**Concomitant diseases**	24 (24.7)	41 (35.0)	0.1351
**Extraintestinal manifestations**	30 (30.9)	22 (18.8)	0.0542

* Systemic steroid therapy included: methylprednisolone, prednisone, hydrocortisone. ** Immunosuppressive and anti-inflammatory agents included: azathioprine, methotrexate, mercaptopurine, cyclosporine, mycophenolate mofetil, tacrolimus. *** Biological agents included: infliximab, adalimumab, golimumab, vedolizumab. **** Only surgery related to IBD-specific problems (e.g., colectomy, resection, fistula, perforation, abscess) was included.

**Table 2:** Clinical characteristics of patients with Crohn’s disease depending on the season of diagnosis.

Variables Median (IQR) or *n* (%)	Winter *n* = 88	Spring *n* = 87	Summer *n* = 125	Autumn *n* = 106	*p* Value
**Age**					
At diagnosis	11.9 (9.3–13.7)	12.4 (10.3–14.7)	12.7 (9.0–13.9)	12.7 (10.6–15.5)	0.4013
At worst flare	12.7 (10.8–14.5)	14.6 (12.5–15.8)	13.7 (12.1–16.1)	13.6 (10.8–15.9)	0.2557
**Selected biochemical parameters**					
CRP at diagnosis [mg/L]	8.3 (2.4–21.0)	12.8 (2.3–29.8)	15.1 (1.8–32.2)	13.8 (1.9–23.6)	0.7752
CRP at worst flare [mg/L]	12.5 (2.6–26.7)	13.1 (5.1–42.6)	12.9 (2.1–32.5)	14.4 (1.9–34.0)	0.7838
Albumin level at diagnosis [g/dL]	**4.1 (3.8–4.4)**	**3.8 (3.5–4.0)**	**3.7 (3.4–4.2)**	**4.0 (3.8–4.3)**	**0.0164**
Albumin level at worst flare [g/dL]	3.9 (3.6–4.4)	3.9 (3.6–4.0)	3.9 (3.4–4.2)	3.9 (3.7–4.3)	0.7368
**Disease activity scales**					
PCDAI at diagnosis	24 (19–36)	35 (25–50)	35 (25–49)	33 (25–48)	0.0600
PCDAI at worst flare	31 (21–53)	46 (30–53)	45 (30–55)	40 (30–53)	0.1839
**Treatment**					
Number of patients on systemic steroids *	20 (51.3)	27 (55.1)	40 (57.1)	28 (49.1)	0.8158
Number of courses of steroid treatment	1 (0–1)	1 (1–2)	1 (0–2)	1 (0–2)	0.6480
Number of patients receiving immunosuppressive treatment **	29 (74.4)	39 (79.6)	56 (80.0)	44 (77.2)	0.9056
Number of immunosuppressants	1 (0–1)	1 (1–1)	1 (1–1)	1 (1–1)	0.7221
Time to first dose of immune-suppressive treatment [months]	0.3 (0.0–4.0)	3.0 (0.0–14.0)	2.4 (0.0–12.3)	1.0 (0.0–5.7)	0.3522
Age at first intake of immun0-suppressive treatment [years]	11.8 (9.2–13.9)	13.1 (10.5–14.9)	12.7 (9.8–14.3)	13.4 (11.2–15.6)	0.2298
Number of patients receiving biological therapy ***	15 (38.5)	29 (59.2)	39 (55.7)	24 (42.1)	0.1087
Number of biological agents	0 (0–1)	1 (0–1)	1 (0–1)	0 (0–1)	0.0700
Time to first dose of biological treatment [months]	19.0 (11.0–38.7)	13.3 (5.1–25.5)	13.8 (7.9–25.3)	12.1 (2.0–32.0)	0.7621
Age at first biological treatment	13.7 (10.3–15.1)	14.2 (12.4–15.3)	13.1 (12.0–14.9)	14.1 (10.8–16.2)	0.8263
Number of patients who had operative treatment ****	3 (7.7)	10 (20.4)	7 (10.0)	9 (15.8)	0.2486
Age at first surgery [years]	14.3 (13.3–15.7)	14.6 (13.9–15.6)	13.5 (11.4–14.9)	14.6 (9.4–16.8)	0.7807
Time to first surgery [months]	27.0 (7.9–56.4)	11.8 (1.5–32.1)	29.1 (0.0–43.5)	8.0 (3.0–43.0)	0.8806
**Hospitalisations (if duration ≥1 years)**					
Hospitalisations for relapse (per 1 year)	0.3 (0.2–0.8)	0.6 (0.3–0.9)	0.5 (0.2–0.9)	0.5 (0.3–0.7)	0.5458
Days of hospitalisation for relapse (per 1 year)	2.2 (1.0–5.7)	4.7 (1.6–7.1)	4.9 (1.2–12.7)	3.6 (1.6–6.6)	0.1454
Relapses from diagnosis (per 1 year)	0.3 (0.2–0.8)	0.7 (0.5–1.0)	0.4 (0.1–0.9)	0.5 (0.3–0.7)	0.0697
Severe relapses from diagnosis (per 1 year)	0.1 (0.0–0.3)	0.3 (0.0–0.5)	0.1 (0.0–0.5)	0.3 (0.0–0.4)	0.5402
**Concomitant diseases**	**6 (15.4)**	**17 (34.7)**	**16 (22.9)**	**26 (45.6)**	**0.0052**
**Extraintestinal manifestations**	**4 (10.3)**	**10 (20.4)**	**26 (37.1)**	**13 (22.8)**	**0.0128**

* Systemic steroid therapy included: methylprednisolone, prednisone, hydrocortisone. ** Immunosuppressive and anti-inflammatory agents included: azathioprine, methotrexate, mercaptopurine, cyclosporine, mycophenolate mofetil, tacrolimus. *** Biological agents included: infliximab, adalimumab, golimumab, vedolizumab. **** Only surgery related to IBD-specific problems (e.g., colectomy, resection, fistula, perforation, abscess) was included.

**Table 3:** Clinical characteristics of patients with Crohn’s disease in periods with typically highest and lowest vitamin D status and insolation.

Variables Median (IQR) or *n* (%)	25(OH)D Concentration		*p* Value	Insolation		*p* Value
	Low *n* = 120	High *n* = 135		Low *n* = 137	High *n* = 142	
**Age**						
At diagnosis	12.5 (10.3–14.2)	12.6 (10.1–14.7)	0.6533	12.8 (10.4–14.2)	12.7 (9.0–14.3)	0.6079
At worst flare	14.0 (12.0–15.7)	13.3 (11.5–15.3)	0.3805	13.6 (11.2–16.0)	13.7 (12.1–16.1)	0.6142
**Selected biochemical parameters**						
CRP at diagnosis [mg/L]	12.0 (3.3–29.8)	13.4 (1.1–32.2)	0.8877	12.5 (3.4–22.0)	14.0 (1.5–31.8)	0.7241
CRP at worst flare [mg/L]	13.7 (3.5–31.5)	9.0 (1.3–27.0)	0.1991	14.4 (3.4–31.2)	12.3 (2.9–36.0)	0.6634
Albumin level at diagnosis [g/dL]	3.9 (3.7–4.2)	3.8 (3.4–4.2)	0.2748	**4.1 (3.8–4.4)**	**3.7 (3.4–4.2)**	**0.0036**
Albumin level at worst flare [g/dL]	3.9 (3.6–4.2)	3.9 (3.5–4.2)	0.6546	3.9 (3.6–4.3)	3.9 (3.4–4.2)	0.3650
**Disease activity scales**						
PCDAI at diagnosis	35 (23–50)	37 (25–50)	0.2871	**29 (20–38)**	**30 (25–48)**	**0.0481**
PCDAI at worst flare	43 (30–53)	48 (30–55)	0.3964	35 (23–53)	43 (30–55)	0.1310
**Treatment**						
Number of patients on systemic steroids *	35 (53.0)	36 (51.4)	0.8654	33 (53.2)	46 (56.8)	0.7354
Number of courses of steroid treatment	1 (0–1)	1 (0–2)	0.5929	1 (0–1)	1 (0–2)	0.2950
Number of patients receiving immunosuppressive treatment **	51 (77.3)	56 (80.0)	0.8345	45 (72.6)	64 (79.0)	0.4298
Number of immunosuppressants	1 (1–1)	1 (1–1)	0.4111	1 (0–1)	1 (1–1)	0.2156
Time to first dose of immunosuppressive treatment [months]	1.6 (0.0–8.7)	1.6 (0.0–5.7)	0.9169	0.6 (0.0–4.0)	2.7 (0.0–12.0)	0.1932
Age at first intake of immunosuppressive treatment [years]	12.8 (10.4–14.9)	12.7 (10.7–15.0)	0.8516	12.9 (10.1–15.3)	12.8 (9.8–14.3)	0.6103
Number of patients receiving biological therapy ***	33 (50.0)	33 (47.1)	0.8638	25 (40.3)	46 (56.8)	0.0637
Number of biological agents	0 (0–1)	0 (0–1)	0.9368	**0 (0–1)**	**1 (0–1)**	**0.0220**
Time to first dose of biological treatment [months]	19.4 (5.3–29.4)	11.6 (3.2–28.0)	0.2949	15.5 (5.6–26.7)	12.4 (7.0–22.5)	0.4316
Age at first biological treatment	14.2 (12.4–15.0)	13.1 (10.8–15.0)	0.3303	14.7 (12.7–16.2)	13.4 (11.7–15.2)	0.2431
Number of patients who had operative treatment ****	11 (16.7)	10 (14.3)	0.8135	6 (9.7)	9 (11.1)	1.0000
Age at first surgery [years]	14.6 (14.3–15.7)	13.5 (9.4–16.7)	0.2178	15.6 (13.8–16.8)	13.4 (12.3–14.5)	0.0729
Time to first surgery [months]	17.3 (3.0–41.1)	8.0 (0.0–38.6)	0.7121	27.0 (7.9–56.4)	28.2 (6.0–39.3)	0.8262
**Hospitalisations (if duration ≥1 years)**						
Hospitalisations for relapse (per 1 year)	0.5 (0.2–0.8)	0.6 (0.4–0.9)	0.1048	0.3 (0.2–0.8)	0.5 (0.2–1.0)	0.3501
Days of hospitalisation for relapse (per 1 year)	**3.04 (1.17–5.82)**	**5.2 (2.6–8.4)**	**0.0288**	**2.4 (1.0–6.0)**	**5.1 (1.8–13.1)**	**0.0188**
Relapses from diagnosis (per 1 year)	0.5 (0.2–0.9)	0.6 (0.3–0.9)	0.6117	0.3 (0.2–0.8)	0.5 (0.1–1.1)	0.2844
Severe relapses from diagnosis (per 1 year)	0.2 (0.0–0.5)	0.3 (0.0–0.6)	0.5972	0.2 (0.0–0.3)	0.2 (0.0–0.5)	0.2844
**Concomitant diseases**	21 (31.8)	24 (34.3)	0.8557	16 (25.8)	18 (22.2)	0.6932
**Extraintestinal manifestations**	**11 (16.7%)**	**22 (31.4%)**	**0.0485**	**8 (12.9%)**	**29 (35.8%)**	**0.0020**

* Systemic steroid therapy included: methylprednisolone, prednisone, hydrocortisone. ** Immunosuppressive and anti-inflammatory agents included: azathioprine, methotrexate, mercaptopurine, cyclosporine, mycophenolate mofetil, tacrolimus. *** Biological agents included: infliximab, adalimumab, golimumab, vedolizumab. **** Only surgery related to IBD-specific problems (e.g., colectomy, resection, fistula, perforation, abscess) was included.

## Supplementary Materials

The following are available online at https://www.mdpi.com/2077-0383/9/12/3957/s1, Table S1: Epidemiological patient characteristics, Table S2: Clinical characteristics of patients with inflammatory bowel diseases depending on insolation at diagnosis, Table S3: Clinical characteristics of patients with ulcerative colitis depending on insolation, Table S4: Clinical characteristics of patients with inflammatory bowel diseases depending on the season of diagnosis, Table S5: Clinical characteristics of patients with ulcerative colitis depending on the season of diagnosis.
