# Supplementary material for: Insolation and Disease Severity in Paediatric Inflammatory Bowel Disease—A Multi-Centre Cross-Sectional Study
Source: J Clin Med. 2020 Dec 7;9(12):3957. doi: 10.3390/jcm9123957 (PMC7762204; doi:10.3390/jcm9123957)
Supplement: Supplementary file 1 [file jcm-09-03957-s001.pdf]

Supplementary Table 1. Epidemiological patient characteristics.

Variables<br>Median (IQR) or n (%)	n	Crohn's disease	Ulcerative colitis	p value
Age [y]				
At inclusion	397	15.18 (13.38-17.05)	15.09 (11.67-16.76)	0.038
At diagnosis	404	12.58 (10.02-14.32)	12.14 (7.80-15.00)	0.328
At worst flare	355	13.65 (11.54-15.84)	13.76 (10.07-15.87)	0.236
Duration of the disease [y]	390	2.23 (0.80-4.24)	1.88 (0.35-3.91)	0.249
Body weight at diagnosis [kg]	387	38.6 (27.1-51.0)	40.0 (27.3-53.7)	0.597
Body weight at diagnosis [z score]	383	-0.82 ([-1.39]-[-0.03]	-0.51 ([-1.14]-0.22)	0.003
Body height at diagnosis [cm]	382	151.0 (137.5-164.5)	151.0 (130.0-168.5)	0.911
Body height at diagnosis [z score]	378	-0.37 ([-1.29]-0.45)	0.06 ([-0.68]-0.82]	<0.001
Body mass index at diagnosis [kg/m^2]	382	16.6 (14.5-18.5)	17.3 (15.4-19.3)	0.024
Body mass index at diagnosis [z score]	378	-0.79 ([-1.48]-[-0.02])	-0.49 ([-1.00]-0.15)	0.006
Body weight at worst flare [kg]	345	42.7 (31.0-53.0)	46.9 (31.6-56.9)	0.367
Body weight at worst flare [z score]	339	-0.94 ([-1.45]-[-0.16])	-0.55 ([-0.99]-0.30)	<0.001
Body height at worst flare [cm]	345	158.0 (145.0-168.0)	158.8 (140.0-171.8)	0.900
Body height at worst flare [z score]	340	-0.43 ([-1.31]-0.32)	-0.03 ([-0.67]-0.80)	<0.001
Body mass index at worst flare [kg/m^2]	341	17.0 (15.1-19.5)	17.5 (15.8-20.6)	0.053
Body mass index at worst flare [z score]	337	-0.94 ([-1.46]-0.04)	-0.65 ([-1.12]-0.16	0.008

Supplementary Table 2. Clinical characteristics of patients with inflammatory bowel diseases depending on insolation at diagnosis

**Variables**<br>**Median (IQR) or n (%)**	**Insolation**		**p value**
	**>3 kWh/m^2/day**<br>(April–August)	**<3 kWh/m^2/day**<br>(September–March)	
**Age**			
At diagnosis	12.6 (8.7-14.6)	12.2 (9.3-14.9)	0.9709
At worst flare	13.9 (11.4-16.1)	13.5 (10.6-15.7)	0.2654
**Selected biochemical parameters**			
CRP at diagnosis [mg/l]	8.1 (1.0-25.6)	4.5 (0.8-17.6)	0.0970
CRP at worst flare [mg/l]	6.9 (1.8-29.8)	5.2 (1.0-23.3)	0.3499
Albumin level at diagnosis [g/dl]	4.0 (3.5-4.3)	4.1 (3.7-4.4)	0.0866
Albumin level at worst flare [g/dl]	4.0 (3.5-4.3)	4.0 (3.7-4.4)	0.2171
**Treatment**			
Number of patients on systemic steroids*	117 (65.7)	135 (59.7)	0.2552

Number of courses of steroid treatment	1 (1-2)	1 (0-2)	0.3821
Number of patients receiving immunosuppressive treatment**	123 (69.5)	156 (69.0)	1.0000
Number of immunosuppressants	1 (0-1)	1 (0-1)	0.8271
Time to first dose of immunosuppressive treatment [months]	2.9 (0.0-12.0)	2.0 (0.0-7.2)	0.2494
Age at first intake of immunosuppressive treatment [years]	12.8 (9.4-14.6)	12.4 (9.3-14.7)	0.7461
Number of patients receiving biological therapy***	**81 (45.5)**	**74 (32.7)**	**0.0100**
Number of biological agents	**0 (0-1)**	**0 (0-1)**	**0.0030**[1]
Time from diagnosis to the first dose of biological treatment [months]	12.0 (6.1-26.8)	15.8 (8.0-28.4)	0.3491
Age at first biological treatment	13.3 (10.7-15.3)	13.5 (10.4-15.4)	0.8362
Number of patients who had operative treatment****	16 (9.0)	17 (7.5)	0.5897
Age at first surgery [years]	14.2 (13.2-15.6)	14.4 (10.0-15.6)	0.6907
Time to first surgery [months]	23.1 (0.0-35.0)	16.7 (3.0-43.0)	0.6330
**Hospitalisations (if duration ≥1 years)**			
Hospitalisations for relapse (per 1 year)	0.6 (0.3-1.0)	0.6 (0.3-1.0)	0.8210
Days of hospitalisation for relapse (per 1 year)	4.6 (1.8-8.7)	4.5 (1.3-7.6)	0.3152
Relapses from diagnosis (per 1 year)	0.5 (0.3-1.1)	0.6 (0.2-1.0)	0.4411
Severe relapses from diagnosis (per 1 year)	0.1 (0.0-0.5)	0.2 (0.0-0.4)	0.9244
**Concomitant diseases**	52 (29.2)	81 (35.8)	0.1672
**Extraintestinal manifestations**	45 (25.3)	44 (19.5)	0.1839

* Systemic steroid therapy included: methylprednisolone, prednisone, hydrocortisone.

** Immunosuppressive and anti-inflammatory agents included: azathioprine, methotrexate, mercaptopurine, cyclosporine, mycophenolate mofetil, tacrolimus.

*** Biological agents included: infliximab, adalimumab, golimumab, vedolizumab.

**** Only surgery related to IBD-specific problems (e. g. colectomy, resection, fistula, perforation, abscess) was included.

[1] insolation >3 vs. <3 [kWh/m²/day]: no biologics: 54.5% vs. 67.7%, one agent: 36.0% vs. 28.8%, two agents: 8.4% vs. 3.1%, three agents: 1.1% vs. 0.4%.

Supplementary Table 3. Clinical characteristics of patients with ulcerative colitis depending on insolation.

Variables	Insolation		p
**Median (IQR) or n (%)**	**>3 kWh/m²/day**	**<3 kWh/m²/day**	**value**

	(April–August)	(September–March)	
**Age**			
At diagnosis	12.4 (7.9-14.6)	12.0 (7.9-15.1)	0.9330
At worst flare	13.9 (10.2-15.8)	13.2 (10.1-15.7)	0.7268
**Selected biochemical parameters**			
CRP at diagnosis [mg/l]	2.0 (0.7-13.4)	2.3 (0.4-7.9)	0.3511
CRP at worst flare [mg/l]	2.7 (0.8-14.2)	2.4 (0.5-11.2)	0.4806
Albumin level at diagnosis [g/dl]	4.1 (3.7-4.4)	4.2 (3.5-4.4)	0.6148
Albumin level at worst flare [g/dl]	4.1 (3.6-4.4)	4.2 (3.9-4.4)	0.7147
**Disease activity scales**			
PUCAI at diagnosis	45 (25-55)	48 (30-60)	0.1788
PUCAI at worst flare	55 (38-65)	50 (35-65)	0.2620
**Treatment**			
Number of patients on systemic steroids*	59 (72.8)	78 (71.6)	0.8715
Number of courses of steroid treatment	1 (1-2)	1 (1-2)	0.9810
Number of patients receiving immunosuppressive treatment**	46 (57.5)	65 (59.6)	0.8812
Number of immunosuppressants	1 (0-1)	1 (0-1)	0.7802
Time to first dose of immunosuppressive treatment [months]	4.3 (0.8-12.5)	2.5 (0.0-9.2)	0.1409
Age at first intake of immunosuppressive treatment [years]	11.9 (8.1-14.7)	11.5 (7.0-14.6)	0.5318
Number of patients receiving biological therapy***	25 (30.9)	23 (21.2)	0.1327
Number of biological agents	0 (0-1)	0 (0-0)	0.1429
Time to first dose of biological treatment [months]	17.0 (8.6-29.1)	14.0 (8.0-25.2)	0.5094
Age at first biological treatment	11.6 (7.2-15.3)	13.1 (6.6-15.5)	0.9068
Number of patients who had operative treatment****	2 (2.4)	2 (1.8)	1.0000
Age at first surgery [years]	14.8 (5.9-17.1)	10.0 (8.2-13.0)	0.8597
Time to first surgery [months]	16.8 (5.0-28.7)	16.7 (0.9-37.4)	0.7728
**Hospitalisations (if duration ≥1 years)**			
Hospitalisations for relapse (per 1 year)	0.6 (0.3-1.0)	0.7 (0.3-1.6)	0.4845
Days of hospitalisation for relapse (per 1 year)	4.6 (1.9-6.9)	6.2 (1.3-10.5)	0.4617
Relapses from diagnosis (per 1 year)	0.6 (0.3-1.2)	0.7 (0.2-1.3)	0.8858

Severe relapses from diagnosis (per 1 year)	0.1 (0.0-0.6)	0.1 (0.0-0.5)	0.7079
**Concomitant diseases**	28 (34.6)	40 (36.7)	0.8785
**Extraintestinal manifestations**	15 (18.5)	22 (20.2)	0.8540

* Systemic steroid therapy included: methylprednisolone, prednisone, hydrocortisone.

** Immunosuppressive and anti-inflammatory agents included: azathioprine, methotrexate, mercaptopurine, cyclosporine, mycophenolate mofetil, tacrolimus.

*** Biological agents included: infliximab, adalimumab, golimumab, vedolizumab.

**** Only surgery related to IBD-specific problems (e. g. colectomy, resection, fistula, perforation, abscess) was included.

Supplementary Table 4. Clinical characteristics of patients with inflammatory bowel diseases depending on the season of diagnosis.

Variables<br>Median (IQR) or n (%)	Winter<br>n=88	Spring<br>n=87	Summer<br>n=125	Autumn<br>n=106	p value
**Age**					
At diagnosis	**11.4 (7.9-13.8)**	**12.5 (9.8-14.7)**	**12.6 (8.8-14.4)**	**13.0 (10.1-15.4)**	**0.0477**[1]
At worst flare	12.1 (10.2-15.0)	13.9 (11.5-15.8)	13.9 (11.6-16.1)	13.7 (10.6-15.9)	0.0880
**Selected biochemical parameters**					
CRP at diagnosis [mg/l]	3.8 (1.0-12.9)	8.4 (1.1-23.5)	7.2 (1.0-24.0)	5.6 (0.7-19.3)	0.3368
CRP at worst flare [mg/l]	4.8 (1.5-20.7)	8.4 (2.3-31.5)	6.5 (1.6-27.2)	5.9 (0.5-32.2)	0.6977
Albumin level at diagnosis [g/dl]	4.10 (3.77-4.36)	3.95 (3.52-4.32)	3.97 (3.50-4.30)	4.08 (3.70-4.40)	0.3080
Albumin level at worst flare [g/dl]	4.10 (3.66-4.41)	3.95 (3.59-4.25)	4.00 (3.50-4.30)	4.00 (3.70-4.40)	0.3098
**Disease activity scales**					
PUCAI at diagnosis	47.5 (35.0-65.0)	45.0 (25.0-57.5)	45.0 (25.0-50.0)	50.0 (25.0-60.0)	0.4714
PUCAI at worst flare	50.0 (35.0-65.0)	50.0 (35.0-65.0)	55.0 (37.5-65.0)	52.5 (40.0-65.0)	0.6833
PCDAI at diagnosis	22.5 (17.5-37.5)	35.0 (25.0-50.0)	35.0 (25.0-48.8)	32.5 (65.0-47.5)	0.0545
PCDAI at worst flare	32.5 (20.0-52.5)	47.5 (30.0-52.5)	45.0 (30.0-55.0)	40.0 (30.0-52.5)	0.2188
**Treatment**					
Number of patients on systemic steroids*	57 (64.8)	57 (65.5)	78 (62.4)	61 (57.5)	0.6520
Number of courses of steroid treatment	1 (0-2)	1 (1-2)	1 (0-2)	1 (0-2)	0.8216
Number of patients receiving	61 (69.3)	64 (73.6)	85 (68.5)	70 (66.0)	0.7297

immunosuppressive treatment**					
Number of immunosuppressants	1 (0-1)	1 (0-1)	1 (0-1)	1 (0-1)	0.9017
Time to first dose of immunosuppressive treatment [months]	1.6 (0.0-7.2)	2.9 (0.0-9.9)	3.0 (0.0-13.6)	1.3 (0.0-5.4)	0.2686
Age at first intake of immunosuppressive treatment [years]	**11.3 (8.0-13.6)**	**13.0 (10.3-14.8)**	**12.7 (9.4-14.4)**	**13.3 (10.7-15.5)**	**0.0300**[2]
Number of patients receiving biological therapy***	**24 (27.3)**	**42 (48.3)**	**54 (42.7)**	**36 (34.0)**	**0.0164**[3]
Number of biological agents	**0 (0-1)**	**0 (0-1)**	**0 (0-1)**	**0 (0-1)**	**0.0189**[4]
Time to first dose of biological treatment [months]	19.3 (12.0-29.9)	13.1 (7.0-25.9)	14.0 (7.1-26.9)	12.1 (3.9-26.0)	0.3529
Age at first biological treatment	12.7 (9.7-14.7)	13.7 (10.9-15.3)	12.9 (10.7-15.2)	14.1 (10.2-15.7)	0.5092
Number of patients who had operative treatment****	4 (4.5)	10 (11.5)	9 (7.2)	10 (9.4)	0.3591
Age at first surgery [years]	11.8 (9.6-14.3)	14.6 (13.9-15.6)	13.8 (11.4-14.9)	15.1 (10.6-16.7)	0.3904
Time to first surgery [months]	27.0 (16.7-37.4)	11.8 (1.5-32.1)	28.7 (5.0-35.0)	7.5 (1.9-40.8)	0.7959
**Hospitalisations (if duration ≥1 years)**					
Hospitalisations for relapse (per 1 year)	0.4 (0.2-1.0)	0.6 (0.3-1.0)	0.5 (0.2-0.9)	0.7 (0.3-1.1)	0.5561
Days of hospitalisation for relapse (per 1 year)	3.1 (1.0-7.8)	4.8 (2.8-8.5)	4.5 (1.4-8.5)	4.8 (1.6-7.5)	0.2788
Relapses from diagnosis (per 1 year)	0.3 (0.1-1.0)	0.7 (0.4-1.0)	0.5 (0.2-1.0)	0.7 (0.3-0.9)	0.0761
Severe relapses from diagnosis (per 1 year)	0.0 (0.0-0.3)	0.3 (0.0-0.6)	0.1 (0.0-0.5)	0.2 (0.0-0.5)	0.2049
**Concomitant diseases*******	**24 (27.3)**	**37 (42.5)**	**32 (25.6)**	**41 (38.7)**	**0.0231**[5]
**Extraintestinal manifestations**	16 (18.2)	18 (20.7)	36 (28.8)	20 (18.9)	0.1889

* Systemic steroid therapy included: methylprednisolone, prednisone, hydrocortisone.

** Immunosuppressive and anti-inflammatory agents included: azathioprine, methotrexate, mercaptopurine, cyclosporine, mycophenolate mofetil, tacrolimus.

*** Biological agents included: infliximab, adalimumab, golimumab, vedolizumab.

**** Only surgery related to IBD-specific problems (e. g. colectomy, resection, fistula, perforation, abscess) was included.

*****e.g. celiac disease, bronchial asthma, obesity, gastroesophageal reflux disease, epilepsy, hypothyroidism.

[1] post hoc comparison: autumn vs. winter p=0.0180 (Bonferroni and Holm)

[2] post hoc comparison: autumn vs. winter p=0.0109 (Bonferroni and Holm)

[3] post hoc comparison: spring vs. winter p=0.0130 (Bonferroni and Holm) and summer vs. winter p=0.0563 (Bonferroni), p=0.0463 (Holm)

[4] post hoc comparison: spring vs. winter p=0.0311 (Bonferroni and Holm) and summer vs. winter p=0.0366 (Bonferroni), p=0.0311 (Holm)

[5] post hoc comparison: spring vs. summer p=0.0300 (Bonferroni and Holm)

Supplementary Table 5. Clinical characteristics of patients with ulcerative colitis depending on the season of diagnosis.

**Variables Median (IQR) or n (%)**	**Winter n=88**	**Spring n=87**	**Summer n=125**	**Autumn n=106**	**p value**
**Age**					
At diagnosis	10.8 (7.0-14.7)	12.5 (8.7-14.6)	12.1 (7.9-14.6)	13.5 (9.4-15.3)	0.2410
At worst flare	11.9 (8.0-15.7)	13.5 (10.9-15.1)	14.6 (10.7-15.9)	14.1 (10.5-16.2)	0.3711
**Selected biochemical parameters**					
CRP at diagnosis [mg/l]	2.3 (0.5-7.9)	1.9 (0.7-13.2)	2.1 (0.7-14.2)	2.6 (0.4-6.6)	0.6873
CRP at worst flare [mg/l]	3.4 (1.2-7.9)	2.6 (0.6-13.4)	2.9 (1.0-16.4)	2.2 (0.4-15.8)	0.8511
Albumin level at diagnosis [g/dl]	4.1 (3.5-4.3)	4.3 (3.8-4.5)	4.0 (3.7-4.4)	4.2 (3.6-4.5)	0.4759
Albumin level at worst flare [g/dl]	4.2 (3.9-4.4)	4.1 (3.6-4.4)	4.0 (3.8-4.4)	4.1 (3.8-4.4)	0.8428
**Disease activity scales**					
PUCAI at diagnosis	48 (35-65)	45 (25-60)	45 (25-50)	50 (25-60)	0.5207
PUCAI at worst flare	50 (35-65)	50 (35-65)	55 (38-65)	53 (40-65)	0.6833
**Treatment**					
Number of patients on systemic steroids*	37 (75.5)	30 (78.9)	38 (69.1)	33 (67.3)	0.5784

Number of courses of steroid treatment	1 (1-2)	1 (1-2)	1 (1-2)	1 (1-2)	0.8440
Number of patients receiving immunosuppressive treatment**	32 (65.3)	25 (65.8)	29 (53.7)	26 (53.1)	0.4123
Number of immunosuppressants	1 (0-1)	1 (0-1)	1 (0-1)	1 (0-1)	0.5952
Time to first dose of immunosuppressive treatment [months]	4.9 (0.3-10.0)	2.7 (1.0-8.1)	4.6 (0.5-28.4)	2.0 (0.0-4.0)	0.1381
Age at first intake of immunosuppressive treatment [years]	10.1 (6.4-13.3)	13.0 (9.1-14.2)	12.0 (8.0-14.9)	12.9 (9.7-14.9)	0.2733
Number of patients receiving biological therapy***	9 (18.4)	13 (34.2)	15 (27.3)	12 (24.5)	0.4021
Number of biological agents	0 (0-0)	0 (0-1)	0 (0-1)	0 (0-0)	0.4547
Time to first dose of biological treatment [months]	23.5 (12.6-29.7)	13.1 (11.0-26.9)	16.5 (6.1-37.3)	11.8 (5.6-16.4)	0.3167
Age at first biological treatment	10.4 (6.3-13.1)	12.5 (7.4-15.3)	11.6 (8.0-15.3)	13.9 (8.3-15.5)	0.6815
Number of patients who had operative treatment****	1 (2.0)	0 (0.0)	2 (3.6)	1 (2.0)	0.6934
Age at first surgery [years]	9.6 (6.8-10.4)		14.8 (5.9-17.1)	15.57260	1.0000
Time to first surgery [months]	27.1 (16.7-37.4)		16.8 (5.0-28.7)	0.85526	1.0000
**Hospitalisations (if duration ≥1 years)**					

Hospitalisations for relapse (per 1 year)	0.6 (0.3-1.3)	0.6 (0.3-2.2)	0.6 (0.3-0.9)	0.7 (0.4-1.7)	0.4690
Days of hospitalisation for relapse (per 1 year)	6.1 (0.6-9.7)	6.5 (3.1-18.5)	4.3 (1.5-5.5)	5.2 (1.8-10.5)	0.4111
Relapses from diagnosis (per 1 year)	0.5 (0.1-1.1)	0.6 (0.3-1.1)	0.6 (0.4-1.2)	0.7 (0.4-1.3)	0.3713
Severe relapses from diagnosis (per 1 year)	0.0 (0.0-0.4)	0.3 (0.0-0.7)	0.0 (0-0.4)	0.1 (0.0-0.5)	0.4006
**Concomitant diseases**	18 (36.7)	20 (52.6)	16 (29.1)	15 (30.6)	0.0971
**Extraintestinal manifestations**	12 (24.5)	8 (21.1)	10 (18.2)	7 (14.3)	0.6255

* Systemic steroid therapy included: methylprednisolone, prednisone, hydrocortisone.

** Immunosuppressive and anti-inflammatory agents included: azathioprine, methotrexate, mercaptopurine, cyclosporine, mycophenolate mofetil, tacrolimus.

*** Biological agents included: infliximab, adalimumab, golimumab, vedolizumab.

**** Only surgery related to IBD-specific problems (e. g. colectomy, resection, fistula, perforation, abscess) was included.
